# Joint Decisions on Production and Pricing with Strategic Consumers for Green Crowdfunding Products

**DOI:** 10.3390/ijerph14091090

**Published:** 2017-09-20

**Authors:** Yuting Chen, Rong Zhang, Bin Liu

**Affiliations:** 1 School of Economics and Management, Shanghai Maritime University, Shanghai 201306, China; 201630710029@stu.shmtu.edu.cn; 2 Research Center of Logistics, Shanghai Maritime University, Shanghai 201306, China; Zhangrong@shmtu.edu.cn

**Keywords:** green crowdfunding product, substitute, pricing discrimination, strategic consumer, myopic consumer

## Abstract

Green crowdfunding is developing as a novel and popular transaction method, which can largely improve the efficiency of raising initial funds and selling innovative green products or services. In this paper, we explore the creator’s joint decisions regarding green crowdfunding products of different quality levels that can sufficiently satisfy consumer preferences. Firstly, considering the characteristics of a green crowdfunding product, we present four pricing strategies when substitutes exist. Then we propose the optimal pricing strategies to maximize the total profit for the creator under different circumstances, facing strategic and myopic consumers. Finally, for the heterogeneity of consumer valuations, we compare the total profits of the four pricing strategies under different values of the substitution coefficient to obtain the optimal pricing and product strategies under the coexistence of strategic and myopic consumers. According to the result, we find that when the fraction of high-type consumers and the gap between high and low valuations is big, or when they are both small, traditional single pricing shows its benefit. However, when the green crowdfunding products are better than their substitute, a line of green products is more likely to be optimal.

## 1. Introduction

Crowdfunding has become an effective way to resolve funding shortages for minor enterprises and self-employed companies. Reward-based crowdfunding, also known as repayment-based crowdfunding, means consumers fund a project on the crowdfunding platform, and get the corresponding product or service after the crowdfunding is successful. Thus funders are regarded as consumers. Crowdfunding is taken as a tool in which the investors can participate in the project with a lower price. Here, crowdfunding is a financing tool, but it is different from the traditional methods, where borrowers are charged a certain interest as their cost when they loan from the banks. However, in crowdfunding, where the funding price (hereafter called the first stage) can be lower than cost, we can see the rewards as the interest paid to the bank. Of course, the first stage price can be higher than cost, and the profit made by the creator is positive at that time. The first and second stage prices (market price) interact with each other, and they are both decision variables. The optimal prices and profit will be derived in Section 2 and Section 3. The global crowdfunding industry started in 2001, followed by tremendous growth recently. The global crowdfunding financing scale has maintained a growth rate of more than 80% from 2010 to reach $19.96 billion in 2016, and it is expected that the scale of the crowdfunding market will reach about 300 billion US dollars in 2025. Kickstarter raised nearly $700 billion to support 77,000 projects in 2015 with an increase of 35% compared with last year.

There are two kinds of green crowdfunding. One is green donation-based crowdfunding, which belongs to non-profit activities and aims at building a green environment and protecting our nature. The other is green reward-based crowdfunding, which is commonly used by companies who sell green products or services. “Green crowdfunding” has been developing in China for several years. With the common popularization of the internet, there are many success cases of green crowdfunding. The first low-carbon crowdfunding project, a methane developed project, based on the Chinese certified emission reduction (CCER) happened in Hong’an County on 24 July 2015. This project raised 200 million yuan within only 5 min and won considerable profit for local farmers through carbon trading. Our work focuses on green reward-based crowdfunding, which mainly sells green crowdfunding products. Green reward-based crowdfunding products mainly refer to renewable products and energy, or products which are made from renewable and recycled materials or energy. For example, there have recently been several successful projects for products made of renewable bark and recycled wood on Kickstarter.

Generally speaking, crowdfunding can be divided into two stages, the funding stage and the sales stage. Some of the projects still make single-pricing decisions as in the currently traditional selling situation. Yet it is often the case that there are similar substitutes on the market, that consumers could choose a substitute on the market or participate in the crowdfunding for the green product. Strategic consumers would judge and weigh the consumer surpluses of the green crowdfunding product and its substitutes in the two stages, and choose a way to maximize the consumer surpluses. Thus, making appropriate decisions at different stages is the key to production and pricing strategies. Therefore, we need to take consumer behavior into account and choose the optimal pricing strategy to maximize the profits for the creator of the green crowdfunding project. Thus our model is also related to the dynamic pricing strategies of substitutes. In addition, consumers have heterogeneous valuations of products. Therefore, the creator had better provide a line of green crowdfunding products with different quality levels to match different consumer preferences.

Our paper provides new insights into quality level differential products in the crowdfunding model. Based on the studies above, we propose an optimal pricing strategy for green crowdfunding products facing the mixed market of strategic and myopic consumers when substitutes exist. In Section 2, we establish a basic model of four different pricing strategies when a creator provides homogeneous products in one project on the crowdfunding platform. Then, we show models that can maximize the profits of the crowdfunding creator in the coexistence of strategic and myopic consumers. In Section 3, we develop a model to explore the optimal pricing and product strategies of the creator facing both strategic and myopic consumers. In Section 4, we make an equilibrium analysis to explore the impacts of the consumers’ valuation distribution on the optimal joint decision.

### Literature Review

With great developments in crowdfunding, a large number of researchers from around the world have been attracted to this field. KD Buysere [1] proposed that crowdfunding is a group behavior in which many individuals contribute their resources to help others or organize an activity. Kuppuswamy and Bayus [2] found that the amount and timing of other sponsors’ contributions had a substitutional effect on potential sponsors’ funding decisions. Calvin Qiu [3] analyzed the biggest crowdfunding platform “Kickstarter” theoretically and experimentally, and proposed that when baseline substitutes exist, the pricing of the crowdfunding and the consumers’ decisions will be influenced. Mollick [4] indicated that the quality of a project was associated with the project success rate. Cumming, D.J. [5] studied renewable energy crowdfunding in 81 countries, and proposed that crowdfunding is more common in countries with low levels of individualism. Lam, P.T.I. [6] proposed that crowdfunding is an efficient way of obtaining green financing, especially for a renewable and sustainable energy project’s life-cycle, compared with other funding ways. There are also several experts do some researches in green product and pricing decision. (i.e., Lao, K.; Tian, H.; Chen, Y.; Zhao, Q.; Weng, Z.; Fan, J.; Davis, B.C.; Cachon, G.P.; Besanko, D. & Winston, W.L.; Aydin, G.; Agrawal, A. [7,8,9,10,11,12,13,14,15,16,17]) Crowdfunding is also suitable for supporting innovative green technology start-ups. Our paper presents pricing and production decisions for green crowdfunding products. We inherit four basic models of crowdfunding pricing strategies from Ming Hu [18] to resolve the pricing problems of product line design. Furthermore, we consider the optimal decision on product and pricing for green crowdfunding products.

There exist many studies researching consumer behavior when facing substitutes (i.e., Su, X.; Su, X. & Zhang, F. [19,20]). Li He [21] introduced a risk factor into the analysis of consumer behavior. Parlaktrk A K. [22] analyzed the influence of the consumers’ strategies on the intertemporal pricing of multi-products and profits gained by the seller. They thought that in certain conditions, the marketing of substitutes can weaken the disadvantageous influence of the consumers’ strategies. Gongbing Bi [23] researched the dynamic pricing decision of two substitutes, and found that the strategic behavior of consumers would diminish the extra profits gained through dynamic pricing by manufacturers. Heqi Zeng [24] studied the dynamic pricing of substitutes under the coexistence of two different kinds of consumers. They found that the decisions of the strategic and myopic consumers would influence the dynamic pricing of the two products differently. We extend the pricing strategies of substitutes to pricing decisions for green crowdfunding products.

There is much research on product decisions, which is also called product line design, in previous studies. Mussa and Rosen [25] and Moorthy [26] studied how firms can use price discrimination to facilitate consumer self-selection between vertically differential products. Desai [27] studied the product line design based on the extent of horizontal differentiation. According to this research, we developed a model to explore the optimal decision of crowdfunding when the creator would provide products of differential quality levels. Liang Guo [28] endogenized consumers’ knowledge of their preferences so that the firm uses the product line not only to increase consumer surplus but also to strategically influence consumer valuations of the product quality. The product line design is extended into green reward-based crowdfunding in this paper.

## 2. Basic Model

Crowdfunding platforms, such as Kickstarter, Jingdong and Taobao crowdfunding, all require project creators to set the prices of products and goals “*T*” of their crowdfunding. The project will succeed only if the fund reaches the goal “*T*”. To gain the largest profits, the creator should choose the best pricing strategy. The following content starts with a basic model:

We refer to *A* as the green crowdfunding product and *B* as the substitute. In fact, almost all crowdfunding projects need a large number of consumers. In order to simplify the model, we consider the case where there are only two consumers involved in this project. The creator proposes a project with information about its specific quality and price on the crowdfunding platform. Suppose the two consumers find that this project is divided into two periods, denoted by *t*, *t* = 1, 2. In each period, there is a consumer noticing this project. We denote the consumer who arrives in the period “t” as $A_{t}$, *t* = 1, 2. Different consumers have different valuations of a same product. In this section, we discuss the joint decision of pricing and product strategies when quality level differentiation exists. The quality level of the green crowdfunding product would also influence the valuation of the consumers, that is, the higher quality level, the higher the valuation that the consumer has. The green level will be positively related to the consumers’ valuation. Mohamed M. Mostafa [29] used valuation methods based on log-logistic, log-normal regression models, which revealed that consumers are willing to pay a premium price for green products. Thus we assume that the valuations of consumers follow the two-point distribution below:

$$V_{h}=Q\lambda \delta_{h}\sigma p+kg\;\mathrm{with}\;\mathrm{probability}\;\varphi \alpha$$

$$V_{l}=Q\lambda \delta_{l}\sigma p+kg\;\mathrm{with}\;\mathrm{probability}\;\varphi \left(1-\alpha \right)$$

where *g* is for the green level of a green crowdfunding product, where g≥1, and g=1 means the product is an ordinary product, not a green one. *k* is the sensitive coefficient of the green degree to consumers and *Q* is the quality level. *p* is the price of the substitute *B*, δ is the substitution coefficient. Differences of the function and quality between the crowdfunding product *A* and its substitute *B* lead to consumers’ different valuations. $\delta_{i}$ (*I* = *h*, *l*) denotes the ratio of valuations of *A* to *B*. $\delta_{h}p$ denotes a high consumer valuation of *A*, and $\delta_{l}p$ denotes a low consumer evaluation of *A*, which satisfies $\delta_{h}>\delta_{l}>0$. If $\delta_{i}>1$, then *A* is better than *B*, otherwise, if $0<\delta_{i}<1$, then *A* is not as good as *B*. The existence of the substitute *B* in the market decreases the consumers’ willingness to participate in the crowdfunding, which will influence the consumers’ valuations of *A*, and make the sum of probabilities of high and low valuations no longer equal to 1. Suppose that the influence coefficient is φ, where 0≤φ≤1. Then the probability of the consumers’ high and low valuations of *A* are φα and φ(1−α). Considering that the result of the funding and the risk of the product quality can influence the consumers’ valuations of *A*, we set λ as the risk coefficient. Since the consumers can not acquire the green crowdfunding product until the second stage, we introduce a discounting factor σ, to describe the valuations of the consumers.

Similarly, we aim to study the difference between the two consumer valuations $\frac{V_{h}}{V_{l}}$, the corresponding evaluating probability α, as well as their influences on the pricing strategy of the creator. (i.e., how should the creator price the green products when facing consumers with different valuations?) Straightforwardly, we rewrite the two-point distribution of the consumer valuations as:

$$V_{h}=Q\lambda \delta \sigma p+kg\;\mathrm{with}\;\mathrm{probability}\;\varphi \alpha \;$$

$$V_{l}=Q\xi \lambda \delta \sigma p+kg\;\mathrm{with}\;\mathrm{probability}\;\varphi \left(1-\alpha \right)$$

In the equation, $\frac{V_{h}}{V_{l}}=\frac{\delta_{h}}{\delta_{l}}=\frac{1}{\xi }$ is the difference of the consumer valuations, which satisfies 0<ξ<1.

When the quality level of a green crowdfunding product is *Q*, the cost of a unit product is $\frac{Q^{2}}{2}$ (Liang Guo & Juanjuan Zhang [28], Mussa & Rosen [25]). Then, the pricing strategies and the corresponding expected profits can be summarized as follows:

*Margin Strategy* (*H*): In this strategy, the creator set the price at $p^{H}=Q^{H}\lambda \delta \sigma p+kg$, aiming at $T^{H}=2Q^{H}\lambda \delta \sigma p+2kg$. The succeeding probability is $s^{H}={\left(\alpha \varphi \right)}^{2}$, and the expected profit is,

$$\pi^{H}={\left(\alpha \varphi \right)}^{2}\left(T^{H}-2\times \frac{{\left(Q^{H}\right)}^{2}}{2}\right)={\left(\alpha \varphi \right)}^{2}\left(2Q^{H}\lambda \delta \sigma p+2kg-{\left(Q^{H}\right)}^{2}\right)$$

The first order condition of the creator’s problem yields the optimal quality level choices $Q^{H}=\lambda \delta \sigma p$. Then we can get the optimal prices, $p^{H}={\left(\lambda \delta \sigma p\right)}^{2}+kg$. Thus the corresponding expected profit of the creator is $\pi^{H}={\left(\alpha \varphi \right)}^{2}({\left(\lambda \delta \sigma p\right)}^{2}+2kg)$.

*Volume Strategy* (*L*): In this strategy, the creator set the price, $p^{L}=Q^{L}\xi \lambda \delta \sigma p+kg$, and aims at $T^{L}=2Q^{L}\xi \lambda \delta \sigma p+2kg$. The succeeding probability is $s^{L}={\left(\varphi \left(2-\alpha \right)\right)}^{2}$, and the expected profit is

$$\pi^{L}={\left(\alpha \varphi \right)}^{2}\left(T^{L}-2\times \frac{{\left(Q^{L}\right)}^{2}}{2})={\left(\varphi \left(2-\alpha \right)\right)}^{2}(2Q^{L}\xi \lambda \delta \sigma p+2kg-{\left(Q^{L}\right)}^{2}\right)$$

The first order condition ($\frac{\partial \pi^{L}}{\partial Q^{L}}=0$) yields the optimal quality level choice $Q^{L}=\xi \lambda \delta \sigma p$. Then we can get the optimal prices, $p^{L}={\left(\xi \lambda \delta \sigma p\right)}^{2}+2kg$. Thus the corresponding expected profit of the creator is $\pi^{L}={\left(\varphi \left(2-\alpha \right)\right)}^{2}({\left(\xi \lambda \delta \sigma p\right)}^{2}+2kg)$.

*Intertemporal Pricing* (*D*): In this strategy, the creator set different prices for consumers arriving at different periods. We denote $P_{t}$ as the price for the consumers arriving at the period *t*, and *t* = 1, 2. There are two plans:

$$\mathrm{plan}\;\mathrm{one}:\;\{\begin{array}{c} \left(Q_{1}^{D},p_{1}^{D}\right)=\left(Q_{1}^{D},Q_{1}^{D}\lambda \delta \sigma p+kg\right)\\ \left(Q_{2}^{D},p_{2}^{D}\right)=\left(Q_{2}^{D},Q_{2}^{D}\xi \lambda \delta \sigma p+kg\right) \end{array}\;\mathrm{or}$$

$$\mathrm{plan}\;\mathrm{two}:\;\{\begin{array}{c} \left(Q_{1}^{D},p_{1}^{D}\right)=\left(Q_{1}^{D},Q_{1}^{D}\xi \lambda \delta \sigma p+kg\right)\\ \left(Q_{2}^{D},p_{2}^{D}\right)=\left(Q_{2}^{D},Q_{2}^{D}\lambda \delta \sigma p+kg\right) \end{array}$$

The target here is $T^{D}=Q_{1}^{D}\lambda \delta \sigma p+Q_{2}^{D}\xi \lambda \delta \sigma p+2kg$. We take plan one as an example. When $A_{1}$ is willing to pay $Q_{1}^{D}\lambda \delta \sigma p$ for the product, and the corresponding probability is αφ, then no matter whether $A_{2}$ is a higher or lower evaluating consumer, he is willing to pay $Q_{2}^{D}\xi \lambda \delta \sigma p$ for the product, and the probability here is αφ+(1−α)φ=φ. In this case, the succeeding probability is $\alpha \varphi^{2}$, and the expected profit is:

$$\pi^{D}=\alpha \varphi \lambda \left(Q_{1}^{D}\lambda \delta \sigma p+Q_{2}^{D}\xi \lambda \delta \sigma p+2kg-\frac{{\left(Q_{1}^{D}\right)}^{2}}{2}-\frac{{\left(Q_{2}^{D}\right)}^{2}}{2}\right)$$

The optimal quality level can be obtained from the first order condition ($\frac{\partial \pi^{D}}{\partial Q_{1}^{D}}=\frac{\partial \pi^{D}}{\partial Q_{2}^{D}}=0$). The optimal quality level is $Q_{1}^{D}=\lambda \delta \sigma p$ and $Q_{2}^{D}=\xi \lambda \delta \sigma p$. Thus the optimal prices would be derived as follows:

$$\mathrm{plan}\;\mathrm{one}:\;\{\begin{array}{c} \left(Q_{1}^{D},p_{1}^{D}\right)=\left(\lambda \delta \sigma p,{\left(\lambda \delta \sigma p\right)}^{2}+kg\right)\\ \left(Q_{2}^{D},p_{2}^{D}\right)=\left(\xi \lambda \delta \sigma p,{\left(\xi \lambda \delta \sigma p\right)}^{2}+kg\right) \end{array}\;\mathrm{or}$$

$$\mathrm{plan}\;\mathrm{two}:\;\{\begin{array}{c} \left(Q_{1}^{D},p_{1}^{D}\right)=\left(\xi \lambda \delta \sigma p,{\left(\xi \lambda \delta \sigma p\right)}^{2}+kg\right)\\ \left(Q_{2}^{D},p_{2}^{D}\right)=\left(\lambda \delta \sigma p,{\left(\lambda \delta \sigma p\right)}^{2}+kg\right) \end{array},$$

and the corresponding expected profit of the creator is:

$$\pi^{D}=\alpha \varphi \lambda \left({\left(\lambda \delta \sigma p\right)}^{2}+{\left(\xi \lambda \delta \sigma p\right)}^{2}+2kg-\frac{{\left(\lambda \delta \sigma p\right)}^{2}}{2}-\frac{{\left(\xi \lambda \delta \sigma p\right)}^{2}}{2}\right)$$

*Menu Pricing* (*M*): In this strategy, the creator publishes a higher price $p_{h}^{M}$ and a lower price $p_{l}^{M}$, which satisfies $p_{l}^{M}\leq Q_{l}\xi \lambda \sigma \delta p+kg\leq p_{h}^{M}\leq Q_{h}\lambda \sigma \delta p+kg$, and their corresponding quality levels are $Q_{h}^{M}$ and $Q_{l}^{M}$, respectively. Furthermore, the target here is $T^{M}=p_{h}^{M}+p_{l}^{M}+2kg$. It requires at least one customer who pays the higher price. A high-evaluating customer $A_{1}$ would prefer the high-pricing option $p_{h}^{M}$, rather than the low-pricing option $p_{l}^{M}$, if and only if the following incentive compatibility (IC) condition is satisfied:

$$\varphi \alpha \left(Q_{l}^{M}\lambda \delta \sigma p+kg-p_{l}^{M}\right)\leq Q_{h}^{M}\lambda \delta \sigma p+kg-p_{h}^{M}$$

where $p_{l}^{M}\leq Q_{l}\xi \lambda \delta \sigma p+kg$. Clearly optimal prices are reached when both inequalities in the constraints are binding, so that $p_{l}^{M}=Q_{l}\xi \lambda \delta \sigma p+kg$ and $p_{h}^{M}=Q_{h}^{M}\lambda \delta \sigma p-\varphi \alpha \left(\lambda \delta \sigma p-\xi \lambda \delta \sigma p\right)Q_{l}^{M}+kg$. Optimal prices would be expressed as follows:

$$\pi^{M}=\left(1-\varphi^{2}{\left(1-\alpha \right)}^{2}\right)\left(p_{h}^{M}+p_{l}^{M}-\frac{{\left(Q_{h}^{M}\right)}^{2}}{2}-\frac{{\left(Q_{l}^{M}\right)}^{2}}{2}\right)$$

The optimal quality level can be obtained from the first order condition ($\frac{\partial \pi^{M}}{\partial Q_{l}^{M}}=\frac{\partial \pi^{M}}{\partial Q_{l}^{M}}=0$). The optimal quality level is $Q_{h}^{M}=\lambda \delta \sigma p$ and $Q_{l}^{M}=\xi \lambda \delta \sigma p-\varphi \alpha \left(\lambda \delta \sigma p-\xi \lambda \delta \sigma p\right)$.

The optimal quality levels and prices are,

(a) if $1<\frac{1}{\xi }<\frac{1+\varphi \alpha }{\varphi \alpha }$, ($Q_{h}^{M}$,$p_{h}^{M}$) = (λδσp, ${\left(\lambda \delta_{h}p\right)}^{2}-\varphi \alpha \left(\lambda \delta \sigma p-\xi \lambda \delta \sigma p\right)\left(\xi \lambda \delta \sigma p-\varphi \alpha \left(\lambda \delta \sigma p-\xi \lambda \delta \sigma p\right)\right)+kg$), ($Q_{l}^{M}$,$p_{l}^{M}$) = (ξλδσp−φα(λδσp−ξλδσp), ${\left(\xi \lambda \delta \sigma p\right)}^{2}-\varphi \alpha \left(\lambda \delta \sigma p-\xi \lambda \delta \sigma p\right)\xi \lambda \delta \sigma p+kg$). The corresponding expected profit for the creator is $\pi^{M}=\left(1-\varphi^{2}{\left(1-\alpha \right)}^{2}\right)\left({\left(\lambda \delta \sigma p\right)}^{2}-\varphi \alpha \left(\lambda \delta \sigma p-\xi \lambda \delta \sigma p\right)\left(\xi \lambda \delta \sigma p-\varphi \alpha \left(\lambda \delta \sigma p-\xi \lambda \delta \sigma p\right)\right)+{\left(\xi \lambda \delta \sigma p\right)}^{2}-\varphi \alpha \left(\lambda \delta \sigma p-\xi \lambda \delta \sigma p\right)\xi \lambda \delta \sigma p+2kg-\frac{{\left(\lambda \delta \sigma p\right)}^{2}}{2}-\frac{{\left(\xi \lambda \delta \sigma p-\varphi \alpha \left(\lambda \delta \sigma p-\xi \lambda \delta \sigma p\right)\right)}^{2}}{2}\right)$.

(b) if $\frac{1}{\xi }>\frac{1+\varphi \alpha }{\varphi \alpha }$, $(Q_{h}^{M}$,$p_{h}^{M}$) = (λδσp, $Q_{h}^{M}\lambda \delta \sigma p+kg)$, ($Q_{l}^{M}$,$p_{l}^{M})$ = (0, 0). The corresponding expected profit for the creator is $\pi^{M}=\left(1-\varphi^{2}{\left(1-\alpha \right)}^{2}\right)\left({\left(\lambda \delta \sigma p\right)}^{2}+kg-\frac{{\left(\lambda \delta \sigma p\right)}^{2}}{2}\right)=\left(1-\varphi^{2}{\left(1-\alpha \right)}^{2}\right)\left(\frac{{\left(\lambda \delta \sigma p\right)}^{2}}{2}+kg\right)$.

## 3. The Crowdfunding Pricing Strategy Facing Different Types of Consumers

Consumers can be divided into strategic and myopic types. They have different buying behaviors when there are substitutes in the market. In the two-stage model, strategic consumers will weigh the consumer surpluses of different products in both stages, while myopic consumers only compare the consumer surpluses of substitutes in the current stage. In reality, the market is a complex coexistence of myopic and strategic consumers. We suppose one consumer can only buy one product. We inherited the models in Hu [18] and Li, X. [30] to search for the optimal pricing and production strategies to maximize the total profits for the creator when dealing with a complex market of strategic and myopic consumers.

We suppose that the price of the substitute *B* is the same in stage 1 and 2. We assume *u*, the consumer valuation of *B*, obeys a uniform distribution in the range of [0, U]. According to the discussion in Section 2, we deduce the consumer valuations of A and B in stage 1 and 2 as follows: $v_{1A}=\lambda \sigma \delta u+kg$, $v_{2A}=\sigma \delta u+kg$, $v_{1B}=u$, $v_{2B}=\sigma u$

Thus, the consumer surplus of *A* and *B* in different stages are:

$$s_{1A}=\lambda \sigma Q\delta u+kg-p_{1A}$$

$$s_{2A}=\sigma Q\delta u+kg-p_{2A}$$

$$s_{1B}=u-p$$

$$s_{2B}=\sigma u-p$$

We list some above symbols in Table 1.

In the following two parts, we discuss the joint decision facing strategic and myopic consumers, respectively. In the third part, we consider the decision facing both strategic and myopic consumers.

### 3.1. Facing Strategic Consumers

We suppose all consumers are strategic consumers. When strategic consumers face the substitutes, they will weigh the consumer surpluses of different products both in the first and second stage. Therefore, in order to succeed in crowdfunding, the project should attract consumers in the first and second stages. Thus, the consumer surpluses should satisfy:

$$\{\begin{array}{c} s_{1A}>s_{1B}>s_{2B}\\ s_{2A}>s_{1B}>s_{2B} \end{array}$$

Besides, we need to discuss the consumers’ choice of first and second stages separately. Only when $s_{2A}>s_{1A}$, will consumers purchase the green crowdfunding product in the second stage. Similarly, consumers will participate in crowdfunding in the first stage when $s_{1A}>s_{2A}$.

Then we discuss $v_{2A}<v_{1B}$ (when the green product’s valuation in the second stage is lower than the substitute in the first stage) and $v_{2A}>v_{1B}$ separately.

**1.** $v_{2A}\leq v_{1B}$

First, we consider the case that consumers prefer to purchase the green product in the second stage. The consumer surplus will satisfy:

$$\{\begin{array}{c} s_{1A}>s_{1B}>s_{2B}\\ s_{2A}>s_{1B}>s_{2B}\\ s_{2A}>s_{1A} \end{array}$$

It is obvious that when $\frac{p_{2A}-p_{1A}}{\sigma \delta Q\left(1-\lambda \right)}<u<\frac{p_{2A}+kg-p}{\sigma \delta Q-1}$, consumers will choose green product *A* in the second stage.

Thus the probability that consumers will purchase the green product *A* in the second stage is defined as $\beta_{1}=\beta_{2}=\frac{\frac{p_{2A}+kg-p}{\sigma \delta Q-1}-\frac{p_{2A}-p_{1A}}{\delta Q\sigma \left(1-\lambda \right)}}{U}$. The income in the second stage is $N\beta p_{2A}$ (*N* is the total number of consumers, Nβ means the number of green product consumers in the second stage here, N=2).

Similarly, when consumers decide to participate in the crowdfunding in the first stage, the consumer surplus should satisfy:

$$\{\begin{array}{c} s_{1A}>s_{1B}>s_{2B}\\ s_{2A}>s_{1B}>s_{2B}\\ s_{1A}>s_{2A} \end{array}$$

Then we can gain the probability of participating in crowdfunding:

(i) When $\frac{p_{2A}-p_{1A}}{\sigma \delta Q\left(1-\lambda \right)}<\frac{p_{1A}+kg-p}{\lambda \sigma \delta Q-1}$, $\varphi_{1}=\frac{\frac{p_{2A}-p_{1A}}{\sigma \delta Q\left(1-\lambda \right)}-p}{U}$
(ii) When $\frac{p_{2A}-p_{1A}}{\sigma \delta Q\left(1-\lambda \right)}>\frac{p_{1A}+kg-p}{\lambda \sigma \delta Q-1}$, $\varphi_{2}=\frac{\frac{p_{1A}+kg-p}{\lambda \sigma \delta Q-1}-p}{U}$

**2.** $v_{2A}>v_{1B}$

In this case, we should discuss $v_{1A}<v_{1B}$ (when the green product’s valuation in the first stage is lower than the substitute in the first stage) and $v_{1A}>v_{1B}$ separately.

(1) $v_{1A}\leq v_{1B}$

We first consider the case that consumers will prefer to purchasing the green product A in the second stage. The consumer surplus will satisfy:

$$\{\begin{array}{c} s_{1A}>s_{1B}>s_{2B}\\ s_{2A}>s_{1B}>s_{2B}\\ s_{2A}>s_{1A} \end{array}$$

Solving the above inequalities, we find that when $u>\frac{p_{2A}-p_{1A}}{\sigma \delta Q\left(1-\lambda \right)}$ or $u>\frac{p_{2A}-p}{\sigma \delta Q-1}$ consumers will choose green product *A* in the second stage.

Then we discuss the two cases separately:

- When $\frac{p_{2A}-p_{1A}}{\sigma \delta Q\left(1-\lambda \right)}>\frac{p_{2A}+kg-p}{\sigma \delta Q-1}$, the probability of buying the green product in the second stage is $\beta_{3}=\beta_{4}=\frac{U-\frac{p_{2A}-p_{1A}}{\delta Q\sigma \left(1-\lambda \right)}}{U}$
  (i) When $\frac{p_{2A}+kg-p}{\sigma \delta Q-1}<\frac{p_{2A}-p_{1A}}{\sigma \delta Q\left(1-\lambda \right)}<\frac{p_{1A}+kg-p}{\lambda \sigma \delta Q-1}$, the probability of participating in the crowdfunding is $\varphi_{3}=\frac{\frac{p_{2A}-p_{1A}}{\sigma \delta Q\left(1-\lambda \right)}-p}{U}$
  (ii) When $\frac{p_{2A}+kg-p}{\sigma \delta Q-1}<\frac{p_{2A}-p_{1A}}{\sigma \delta Q\left(1-\lambda \right)}$ and $\frac{p_{2A}-p_{1A}}{\sigma \delta Q\left(1-\lambda \right)}>\frac{p_{1A}+kg-p}{\lambda \sigma \delta Q-1}$, the probability of participating in the crowdfunding is $\varphi_{4}=\frac{\frac{p_{1A}+kg-p}{\lambda \sigma \delta Q-1}-p}{U}$
- When $\frac{p_{2A}-p_{1A}}{\sigma \delta Q\left(1-\lambda \right)}<\frac{p_{2A}+kg-p}{\sigma \delta Q-1}$, the probability of buying the green product in the second stage is $\beta_{5}=\beta_{6}=\frac{U-\frac{p_{2A}+kg-p}{\sigma \delta Q-1}}{U}$
  (i) When $\frac{p_{2A}+kg-p}{\sigma \delta Q-1}>\frac{p_{2A}-p_{1A}}{\sigma \delta Q\left(1-\lambda \right)}$ and $\frac{p_{2A}-p_{1A}}{\sigma \delta Q\left(1-\lambda \right)}<\frac{p_{1A}+kg-p}{\lambda \sigma \delta Q-1}$, the probability of participating in the crowdfunding is $\varphi_{5}=\frac{\frac{p_{2A}-p_{1A}}{\sigma \delta Q\left(1-\lambda \right)}-p}{U}$
  (ii) When $\frac{p_{1A}+kg-p}{\lambda \sigma \delta Q-1}<\frac{p_{2A}-p_{1A}}{\sigma \delta Q\left(1-\lambda \right)}<\frac{p_{2A}+kg-p}{\sigma \delta Q-1}$, the probability of participating in the crowdfunding is $\varphi_{6}=\frac{\frac{p_{1A}+kg-p}{\lambda \sigma \delta Q-1}-p}{U}$

(2) $v_{1A}>v_{1B}$

We first consider the case that consumers prefer to purchase the green product *A* in the second stage. The consumer surplus will satisfy:

$$\{\begin{array}{c} s_{1A}>s_{1B}>s_{2B}\\ s_{2A}>s_{1B}>s_{2B}\\ s_{2A}>s_{1A} \end{array}$$

Solving the above inequalities, we find that when $u>\frac{p_{2A}-p_{1A}}{\sigma \delta Q\left(1-\lambda \right)}$ or $u>\frac{p_{2A}+kg-p}{\sigma \delta Q-1}$ consumers will choose green product *A* in the second stage.

Then we discuss the two cases separately:

- When $\frac{p_{2A}-p_{1A}}{\sigma \delta Q\left(1-\lambda \right)}>\frac{p_{2A}+kg-p}{\sigma \delta Q-1}$, the probability of buying the green product in the second stage is $\beta_{7}=\frac{U-\frac{p_{2A}-p_{1A}}{\delta Q\sigma \left(1-\lambda \right)}}{U}$, and the probability of participating in the crowdfunding is $\varphi_{7}=\frac{\frac{p_{2A}-p_{1A}}{\sigma \delta Q\left(1-\lambda \right)}-\frac{p_{1A}+kg-p}{\lambda \sigma \delta Q-1}}{U}$
- When $\frac{p_{2A}-p_{1A}}{\sigma \delta Q\left(1-\lambda \right)}<\frac{p_{2A}-p}{\sigma \delta Q-1}$, $\beta_{8}=\frac{U-\frac{p_{2A}+kg-p}{\sigma \delta Q-1}}{U}$, $\varphi_{8}=\frac{\frac{p_{2A}-p_{1A}}{\sigma \delta Q\left(1-\lambda \right)}-\frac{p_{1A}+kg-p}{\lambda \sigma \delta Q-1}}{U}$

### 3.2. Facing Myopic Consumers

Myopic consumers will purchase the product only when the surplus of the crowdfunding product *A* is larger than that of its substitute *B* in the current stage. Therefore, the pricing of the crowdfunding products in the two stages are independent of each other.

The condition that the consumer attend crowdfunding in the first stage is, $s_{1A}>s_{1B}$

- When $v_{1A}>v_{1B}$, $u>\frac{p_{1A}+kg-p}{\lambda \sigma \delta Q-1}$, the probability of participating in crowdfunding is $\phi_{1}=\frac{U-\frac{p_{1A}+kg-p}{\lambda \sigma \delta Q-1}}{U}$
- When $v_{1A}<v_{1B}$, $u<\frac{p_{1A}+kg-p}{\lambda \sigma \delta Q-1}$, $\phi_{2}=\frac{\frac{p_{1A}+kg-p}{\lambda \sigma \delta Q-1}-p}{U}$
- When $v_{1A}=v_{1B}$, $u=\frac{U}{2}$, $\phi_{3}=\frac{U-\frac{U}{2}}{U}=\frac{1}{2}$

The condition that the consumer will purchase the green product in the second stage is, $s_{2A}>s_{2B}$

- When $v_{2A}>v_{2B}$, $u>\frac{p_{2A}+kg-p}{\sigma \left(\delta Q-1\right)}$, the probability of buying the green product in the second stage is $\gamma_{1}=\frac{U-\frac{p_{2A}+kg-p}{\sigma \left(\delta Q-1\right)}}{U}$
- When $v_{2A}<v_{2B}$, $u<\frac{p_{2A}+kg-p}{\sigma \left(\delta Q-1\right)}$, $\gamma_{2}=\frac{\frac{p_{2A}+kg-p}{\sigma \left(\delta Q-1\right)}-p}{U}$
- When $v_{2A}=v_{2B}$, $u=\frac{U}{2}$, $\gamma_{3}=\frac{U-\frac{U}{2}}{U}=\frac{1}{2}$

### 3.3. Facing Strategic and Myopic Consumers

In reality, the market is a complex coexistence of myopic and strategic consumers. For convenience, we normalize the number of consumers, and set θ as the fraction of strategic consumers, so that the fraction of the myopic consumers is 1−θ. Thus we can derive the probability of choosing a green product in first and second stages:

$$\{\begin{array}{c} \Phi_{i}=\theta \varphi_{i}+\left(1-\theta \right)\phi_{m}\\ \Omega_{j}=\theta \beta_{j}+\left(1-\theta \right)\gamma_{n} \end{array}$$

where θ is the fraction of the strategic consumer, $\Phi_{i}$ is the probability of participating in crowdfunding, and $\Omega_{j}$ is the probability of buying the green product in the second stage.

**Lemma** **1.** *If the creator provides a line of green products when a substitute exists, the total profits for the creator (including the profits in the first and second stages) of the four pricing strategies when facing both strategic and myopic consumers can be summarized as follows:*

$$\Pi_{ij}^{H}\left(p_{1A},p_{2A},Q\right)={\left(\alpha \Phi_{i}\right)}^{2}\left(2Q^{H}\lambda \delta \sigma p+2kg-{\left(Q^{H}\right)}^{2}\right)+2\Omega_{j}(p_{2A}-\frac{{Q^{H}}^{2}}{2})$$

$$\Pi_{ij}^{L}\left(p_{1A},p_{2A},Q\right)={\left(\Phi_{i}\left(2-\alpha \right)\right)}^{2}(2Q^{L}\xi \lambda \delta \sigma p+2kg-{\left(Q^{L}\right)}^{2})+2\Omega_{j}(p_{2A}-\frac{{Q^{L}}^{2}}{2})$$

$$\Pi_{ij}^{D}\left(p_{1A},p_{2A},Q\right)=\alpha \Phi_{i}\lambda \left(Q_{1}^{D}\lambda \delta \sigma p+Q_{2}^{D}\xi \lambda \delta \sigma p+2kg-\frac{{\left(Q_{1}^{D}\right)}^{2}}{2}-\frac{{\left(Q_{2}^{D}\right)}^{2}}{2}\right)+\Omega_{j}(2p_{2A}-\frac{{\left(Q_{1}^{D}\right)}^{2}}{2}-\frac{{\left(Q_{2}^{D}\right)}^{2}}{2})$$

$$\Pi_{ij}^{M}\left(p_{1A},p_{2A},Q\right)=\left(1-\Phi_{i}{}^{2}{\left(1-\alpha \right)}^{2}\right)\left(p_{h}^{M}+p_{l}^{M}-\frac{{\left(Q_{h}^{M}\right)}^{2}}{2}-\frac{{\left(Q_{l}^{M}\right)}^{2}}{2}\right)+\Omega_{j}(2p_{2A}-\frac{{\left(Q_{h}^{M}\right)}^{2}}{2}-\frac{{\left(Q_{l}^{M}\right)}^{2}}{2})$$

$$(\mathrm{where}\;\mathrm{in}\;p_{l}^{M}=Q_{l}\xi \lambda \delta \sigma p+kg,\;p_{h}^{M}=Q_{h}^{M}\lambda \delta \sigma p-\Phi_{i}\alpha \left(\lambda \delta \sigma p-\xi \lambda \delta \sigma p\right)Q_{l}^{M}+kg).$$

Here $\Phi_{i}=\theta \varphi_{i}+\left(1-\theta \right)\phi_{m}$, $\Omega_{j}=\theta \beta_{j}+\left(1-\theta \right)\gamma_{n}$, θ is the fraction of strategic consumers, and i=j=1, 2, 3, 4, 5, 6, 7, 8. *Q* is the quality level, *g* is the green degree, *k* is the sensitive coefficient of the green degree to the consumers. As we have mentioned above, $\varphi_{i}$ and $\beta_{j}$ are the functions of the decision variables ($p_{1A}$, $p_{2A}$ and *Q*).

The optimal profits of the four strategies can be gained from following non-linear programming

$$\underset{p_{1A},p_{2A,}Q}{\max}\Pi_{ij}^{s}$$

$$\{\begin{array}{c} s_{1A}>0\\ s_{2A}>0\\ p_{1A,}\;p_{2A,}Q>0 \end{array}$$

where s=H, L, D, M, i=j=1, 2, 3, 4, 5, 6, 7, 8.

## 4. Equilibrium Analysis

**Figure 1:**
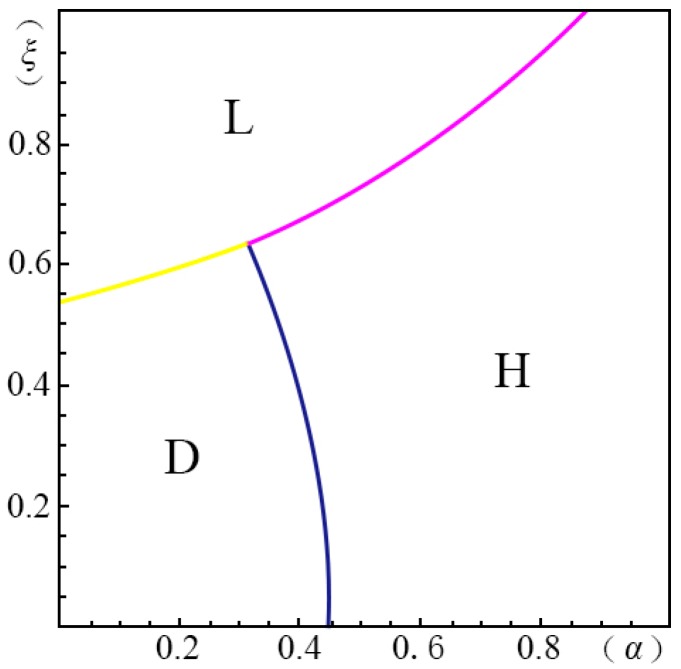
Comparison of the four strategies given $\delta =\frac{1}{2}$ when $\frac{p_{2A}-p_{1A}}{\sigma \delta Q\left(1-\lambda \right)}\leq \frac{p_{1A}+kg-p}{\lambda \sigma \delta Q-1}$.

**Figure 2:**
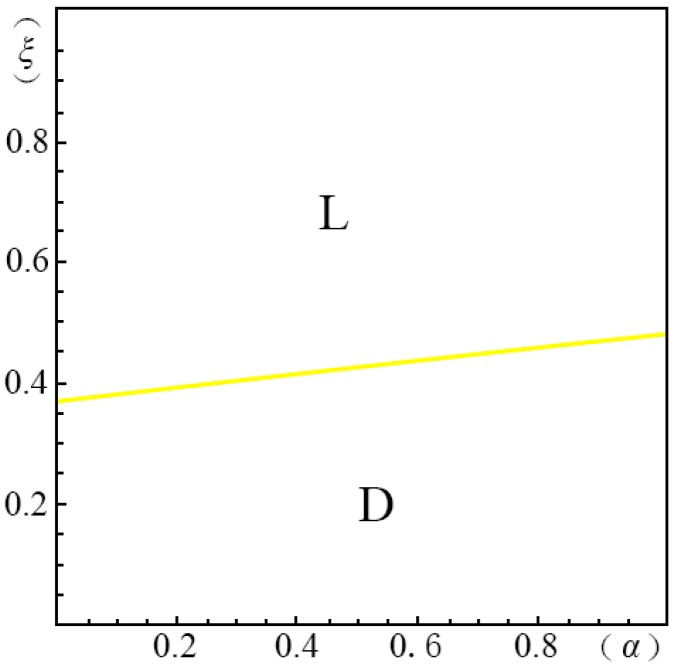
Comparison of the four strategies given δ=1 when $\frac{p_{2A}-p_{1A}}{\sigma \delta Q\left(1-\lambda \right)}\leq \frac{p_{1A}+kg-p}{\lambda \sigma \delta Q-1}$.

**Figure 3:**
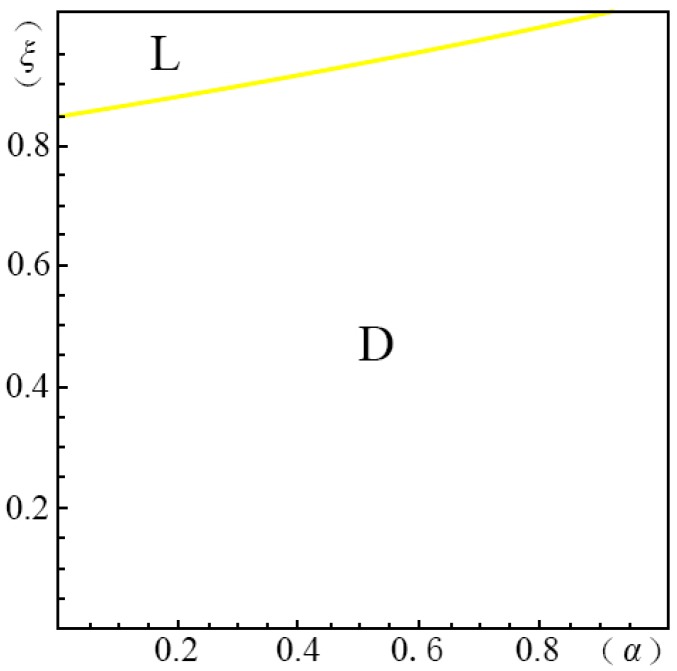
Comparison of the four strategies given $\delta =\frac{1}{2}$ when $\frac{p_{2A}-p_{1A}}{\sigma \delta Q\left(1-\lambda \right)}>\frac{p_{1A}+kg-p}{\lambda \sigma \delta Q-1}$.

**Figure 4:**
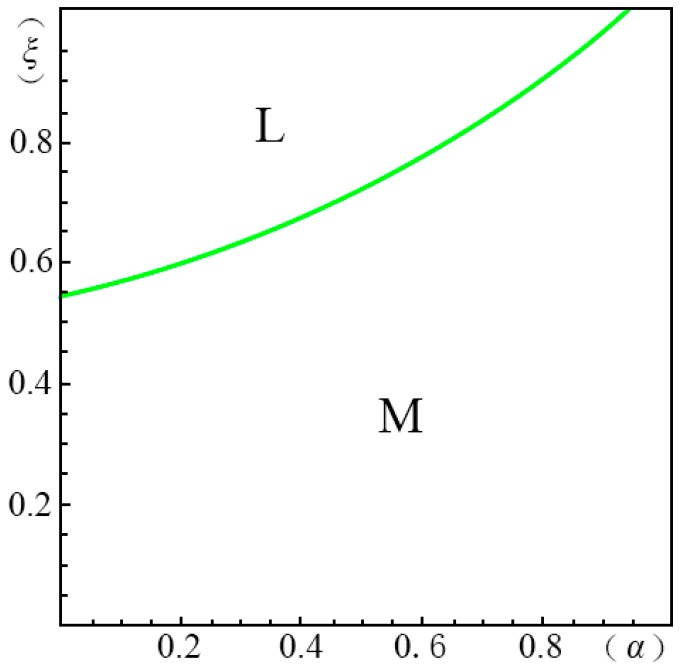
Comparison of the four strategies given δ=2 when $\frac{p_{2A}+kg-p}{\sigma \delta Q-1}\leq \frac{p_{2A}-p_{1A}}{\sigma \delta Q\left(1-\lambda \right)}<\frac{p_{1A}+kg-p}{\lambda \sigma \delta Q-1}.$

**Figure 5:**
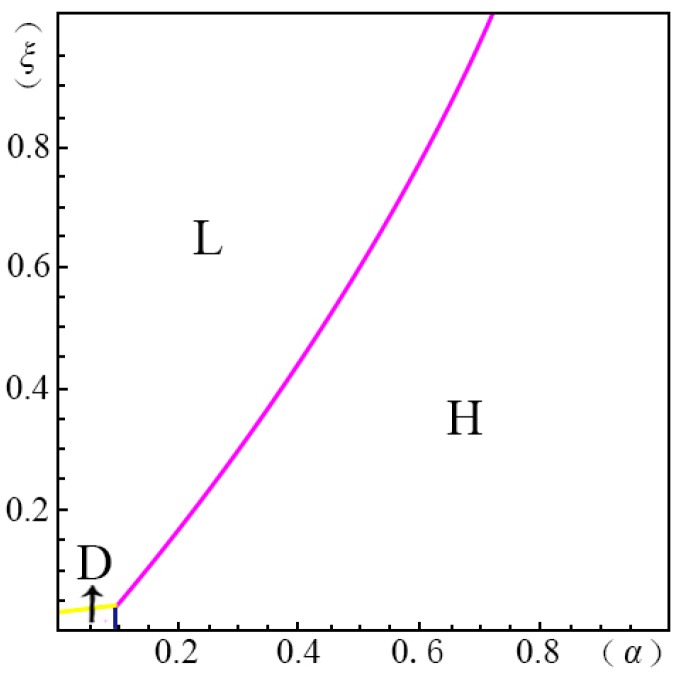
Comparison of the four strategies given δ=2 when $\frac{p_{1A}+kg-p}{\lambda \sigma \delta Q-1}\leq \frac{p_{2A}-p_{1A}}{\sigma \delta Q\left(1-\lambda \right)}<\frac{p_{2A}+kg-p}{\sigma \delta Q-1}$.

**Figure 6:**
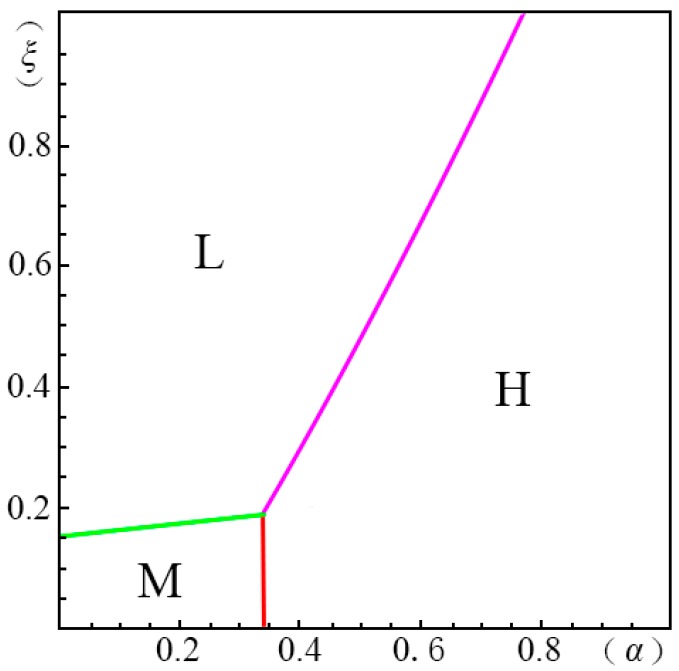
Comparison of the four strategies given δ=3 when $\frac{p_{2A}-p_{1A}}{\sigma \delta Q\left(1-\lambda \right)}>\frac{p_{2A}+kg-p}{\sigma \delta Q-1}$.

**Figure 7:**
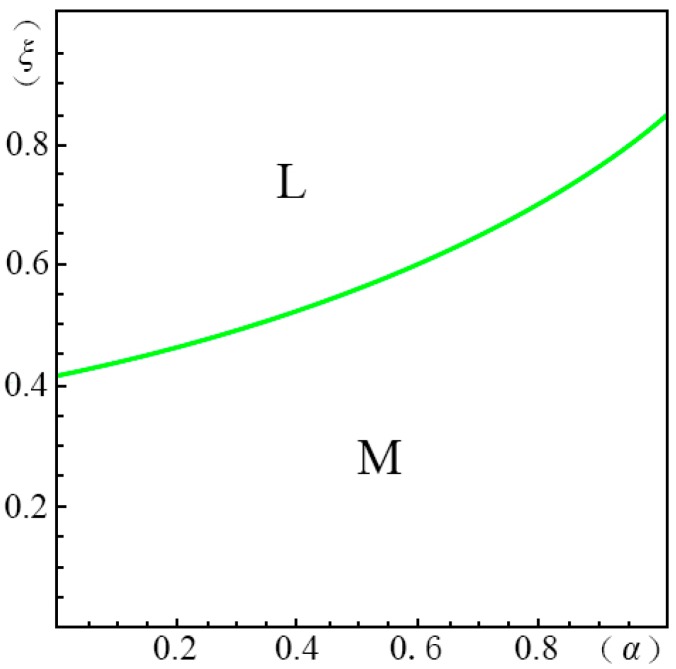
Comparison of the four strategies given δ=3 when $\frac{p_{2A}-p_{1A}}{\sigma \delta Q\left(1-\lambda \right)}<\frac{p_{2A}+kg-p}{\sigma \delta Q-1}$.

To visualize the valuation difference $\frac{V_{h}}{V_{l}}$ of the consumers and show the influence of the pricing probability α on the pricing strategy of the creator, we make the following equilibrium analysis. We suppose the substitute *B* has a unit price (i.e., p=1). The valuation of the substitute *B* by a consumer, *u*, obeys a uniform distribution in the range of [0, U]. We suppose the maximum consumer valuation of *B* is 2 (i.e., U=2). Besides, according to Ming Hu [18], Bi [23], Pan Liu [31] and Jiang [32], we have $\sigma =\frac{4}{5}$, $\lambda =\frac{3}{5}$, $g=\frac{6}{5}$, k=1. We set the substitution coefficient $\delta =\frac{1}{2},\;1,\;2,\;3$ to simulate several cases discussed in the model above. Non-linear programming provides us with the solution for the maximum profits. According to Yossi Aviv [33], we set the fraction of the strategic consumers $\theta =\frac{4}{5}$. By comparing the profits of the four pricing strategies (i.e., $\Pi_{ij}^{H*}$, $\Pi_{ij}^{L*}$, $\Pi_{ij}^{D*}$, $\Pi_{ij}^{M*}$), we define $\xi_{mn}\left(\alpha \right)=\{\xi |\arg\left(\Pi_{ij}^{m*}-\Pi_{ij}^{n*}=0\right)\}$, wherein m,n=H,L,D,M. We demonstrate the results from Figure 1, Figure 2, Figure 3, Figure 4, Figure 5, Figure 6 and Figure 7. The vertical axis represents the ratio of the valuation of low-type to high-type buyers $\xi =\frac{V_{l}}{V_{h}}$; and the horizontal axis represents the fraction of high-type buyers (α) in the market.

**1.** $v_{2A}\leq v_{1B}$

**Proposition** **1.** *Under the condition* $v_{2A}\leq v_{1B}$, *the creator’s optimal strategy is:*

(i) if $\frac{p_{2A}-p_{1A}}{\sigma \delta Q\left(1-\lambda \right)}\leq \frac{p_{1A}+kg-p}{\lambda \sigma \delta Q-1}$,
  when given $\delta =\frac{1}{2}$, as shown in Figure 1:
  (a) *volume strategy* (*L*), if $\xi_{LD}\left(\alpha \right)\leq \xi \leq 1$ and $\xi_{LH}\left(\alpha \right)\leq \xi \leq 1$
  (b) *intertemporal strategy* (*D*), if $\xi_{DH}\left(\alpha \right)\leq \xi \leq \xi_{LD}\left(\alpha \right)$
  (c) *margin strategy* (*H*), if $0\leq \xi \leq \xi_{DH}\left(\alpha \right)$ and $0\leq \xi \leq \xi_{LH}\left(\alpha \right)$
  when given δ=1, as shown in Figure 2:
  (a) *volume strategy* (*L*), if $\xi_{LD}\left(\alpha \right)\leq \xi <1$
  (b) *margin strategy* (*D*), if $0<\xi <\xi_{LD}\left(\alpha \right)$

In case of $\delta =\frac{1}{2}$, shown in Figure 1, menu pricing (*M*) has no advantages. When the fraction of high-type consumers (α) is low and the gap between high and low valuations ($\frac{1}{\xi }$) is large, the *intertemporal strategy* (*D*) can enable the creator to earn maximum profits. On the contrary, it is wise to choose the *margin strategy* (*H*) when the fraction of high-type consumers is large. When the gap between the high and low valuations of consumers ($\frac{1}{\xi }$) is small, then the *volume strategy* (*L*) is the most applicable.

In Figure 2, we can also find that when δ=1, (i.e., when the green crowdfunding product is able to be substituted by its substitute in functions or appearance), only the *volume strategy* (*L*) and *intertemporal strategy* (*D*) are applicable. When the gap between the high and low valuations is large, the *intertemporal strategy* (*D*) is the optimal strategy. In the opposite case, the *volume strategy* (*L*) is more applicable.

(ii) If $\frac{p_{2A}-p_{1A}}{\sigma \delta Q\left(1-\lambda \right)}>\frac{p_{1A}+kg-p}{\lambda \sigma \delta Q-1}$, as show in Figure 3,
  (a) *volume strategy* (*L*), if $\xi_{LD}\left(\alpha \right)\leq \xi <1$
  (b) *intertemporal strategy* (*D*), if $0<\xi <\xi_{LD}\left(\alpha \right)$

In Figure 3 we see that when $\delta =\frac{1}{2}$, only the *volume strategy* (*L*) and *intertemporal strategy* (*D*) are applicable. When the gap between high and low valuations is large and the fraction of high-type consumers is large, the *intertemporal strategy* (*D*) can enable the creator to earn more profits. That is to say that the creators had better choose the *intertemporal strategy* (*D*) if they would like to provide products of differential qualities. Otherwise, the *volume strategy* (*L*) is more applicable.

**2.** $v_{2A}>v_{1B}$

(1) $v_{1A}\leq v_{1B}$

**Proposition** **2.** *Under* $v_{1A}>v_{1B}$ *and* $v_{1A}\leq v_{1B}$, *the creator’s optimal strategy is*

(i) when $\frac{p_{2A}+kg-p}{\sigma \delta Q-1}\leq \frac{p_{2A}-p_{1A}}{\sigma \delta Q\left(1-\lambda \right)}<\frac{p_{1A}+kg-p}{\lambda \sigma \delta Q-1}$, as shown in Figure 4,
  (a) *volume strategy* (*L*), if $\xi_{LM}\left(\alpha \right)\leq \xi <1$;
  (b) *menu strategy* (*M*), if $0\leq \xi <\xi_{LM}\left(\alpha \right)$.
(ii) when $\frac{p_{1A}+kg-p}{\lambda \sigma \delta Q-1}\leq \frac{p_{2A}-p_{1A}}{\sigma \delta Q\left(1-\lambda \right)}<\frac{p_{2A}+kg-p}{\sigma \delta Q-1}$, as shown in Figure 5,
  (a) *volume strategy* (*L*), if $\xi_{LD}\left(\alpha \right)\leq \xi <1$, and $\xi_{LH}\left(\alpha \right)\leq \xi <1$;
  (b) *intertemporal strategy* (*D*), if $\xi_{DH}\left(\alpha \right)\leq \xi <\xi_{LD}\left(\alpha \right)$;
  (c) *margin strategy* (*H*), if $0<\xi <\xi_{LH}\left(\alpha \right)$ and $0<\xi <\xi_{DH}\left(\alpha \right)$;

In case of δ=2, as shown in Figure 4, when the fraction of high-type consumers is high but the gap between the high and low valuations is small, the *volume strategy* (*L*) is the best choice. In Figure 4, we also find that there are no application areas for the *margin strategy* (*H*). Thus, in this case, the creator should be cautious with the pricing decision. Making the price too high will inversely decrease the total profits. When the gap between the high and low valuations is large, the *menu strategy* (*M*) shows its applicability.

As is shown in Figure 5, in the case of $\frac{p_{1A}+kg-p}{\lambda \sigma \delta Q-1}\leq \frac{p_{2A}-p_{1A}}{\sigma \delta Q\left(1-\lambda \right)}<\frac{p_{2A}+kg-p}{\sigma \delta Q-1}$, only when both the fraction of high-type consumers and the gap between the high and low valuations are large is the *margin strategy* (*H*) applicable. When the fraction of high-type consumers increases, the *margin strategy* (*H*) begins to show its advantages in turn. The *Intertemporal strategy* (*D*) only shows its advantages when the rate of high-type consumers and the gap between the high and low valuations is small. Cumming, D.J. [5] has shown that green crowdfunding is more common in developing countries when the oil prices are rising. That is to say, green crowdfunding is more popular when the crowdfunding price is much lower, especially in countries with a lot of low-type consumers. Meanwhile, Kuppuswamy, V. [2] analyzed the USA crowdfunding data from Kickstarter and proposed that backer support will be higher for projects with large goals. Those empirical studies demonstrate our study more effectively.

(2) $v_{1A}>v_{1B}$

**Proposition** **3.** *Under the conditions of* $v_{2A}>v_{1B}$ *and* $v_{1A}>v_{1B}$, *the creator’s optimal strategy is,*

(i) when $\frac{p_{2A}-p_{1A}}{\sigma \delta Q\left(1-\lambda \right)}>\frac{p_{2A}+kg-p}{\sigma \delta Q-1}$, as shown in Figure 6,
  (a) *volume strategy* (*L*), if $\xi_{LM}\left(\alpha \right)\leq \xi <1$ and $\xi_{LH}\left(\alpha \right)\leq \xi <1$;
  (b) *menu strategy* (*M*), if $\xi_{MH}\left(\alpha \right)\leq \xi <\xi_{LM}\left(\alpha \right)$
  (c) *margin strategy* (*H*), if $0<\xi <\xi_{LH}\left(\alpha \right)$ and $0<\xi <\xi_{MH}\left(\alpha \right)$;
(ii) when $\frac{p_{2A}-p_{1A}}{\sigma \delta Q\left(1-\lambda \right)}<\frac{p_{2A}+kg-p}{\sigma \delta Q-1}$, as shown in Figure 7,
  (a) *volume strategy* (*L*), if $\xi_{LM}\left(\alpha \right)\leq \xi <1$;
  (b) *menu strategy* (*M*), if $0\leq \xi <\xi_{LM}\left(\alpha \right)$.

In Figure 6, we find that only when the fraction of the high-type consumers is small and the gap between the high and low valuations is large, is the *menu strategy* (*M*) applicable. In this case, the creator should choose to produce two products of differential quality levels and make two differential prices to satisfy consumer needs. When both the fraction of high-type consumers and the gap between the high and low valuations are high, the *margin strategy* (*H*) is the most applicable.

In Figure 7, the *menu strategy* (*M*) occupies a large application region with a large α and $\frac{1}{\xi }$. In this case, the creator should choose to produce two products of differential quality levels to satisfy the needs of consumers with different valuations. Likewise, when both the fraction of the high-type consumers and the gap between the high and low valuations is small, the *volume strategy* (*L*) begins to show its applicability.

## 5. Exploring the Influence of the Fraction of Strategic Consumers (θ) on the Total Profits

**Figure 8:**
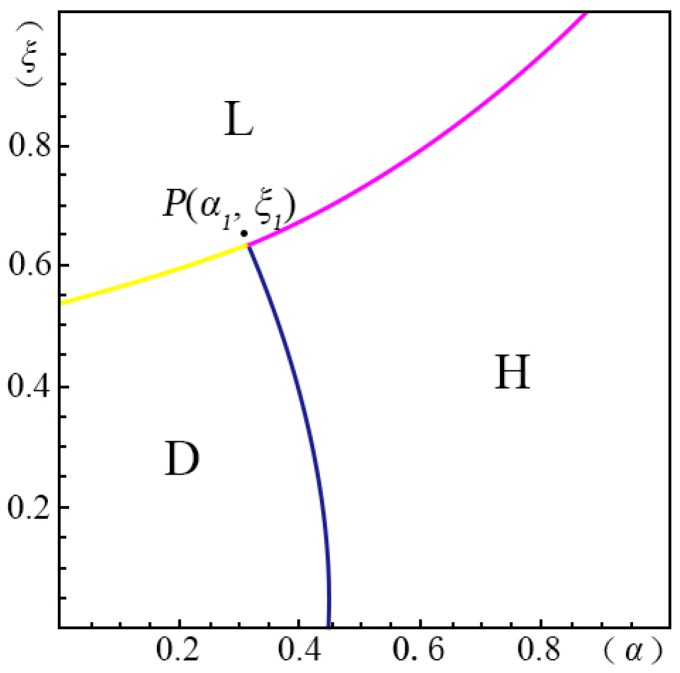
Comparison of the four strategies when $v_{2A}<v_{1B}$ and $\frac{p_{2A}-p_{1A}}{\sigma \delta Q\left(1-\lambda \right)}\leq \frac{p_{1A}-p}{\lambda \sigma \delta Q-1}$.

We take the case of $v_{2A}<v_{1B}$ and $\frac{p_{2A}-p_{1A}}{\sigma \delta Q\left(1-\lambda \right)}\leq \frac{p_{1A}-p}{\lambda \sigma \delta Q-1}$ as an example to explore the influence of the fraction of strategic consumers (θ) on the total profits of the creator, as shown in Figure 8. $P\left(\alpha_{1},\xi_{1}\right)$ is a point in Figure 1. We next explore the behavior of $P\left(\alpha_{1},\xi_{1}\right)$ by changing the fraction of strategic consumers (θ).

**Figure 9:**
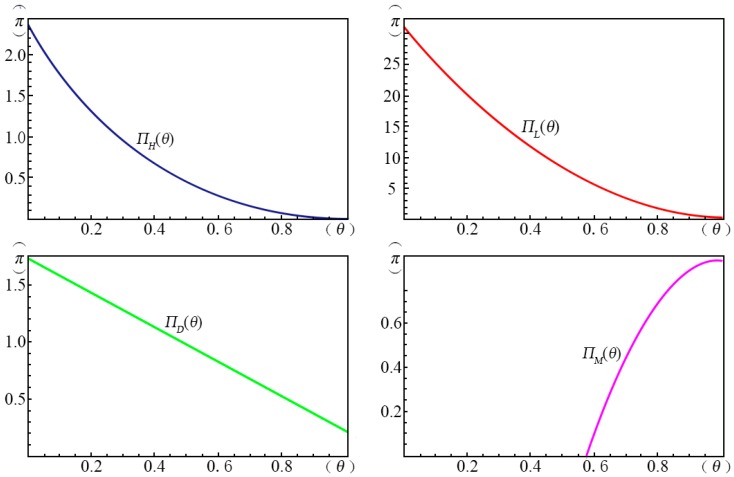
The influence of θ on the profits of the four pricing strategies.

Figure 9 shows the profits of the four pricing strategies (π) as a function of the fraction of strategic consumers (θ) given $\alpha =\alpha_{1}$ and $\xi =\xi_{1}$. We found that when θ is small, the profit of the *margin strategy* (*H*) $\Pi_{H}\left(\theta \right)$ decreases with increasing θ. For the *volume strategy* (*L*), the profit decreases with increasing θ monotonically and the slope is flattened at large θ. For the *intertemporal strategy* (*D*), the profit increases with increasing θ monotonically, while the profit of the *menu strategy* (*M*) increases with increasing θ. However, the profit of the *volume strategy* (*L*) is significantly larger than the others. When θ is below 0.56, the *menu strategy* (*M*) can provide no profit for the creator.

**Figure 10:**
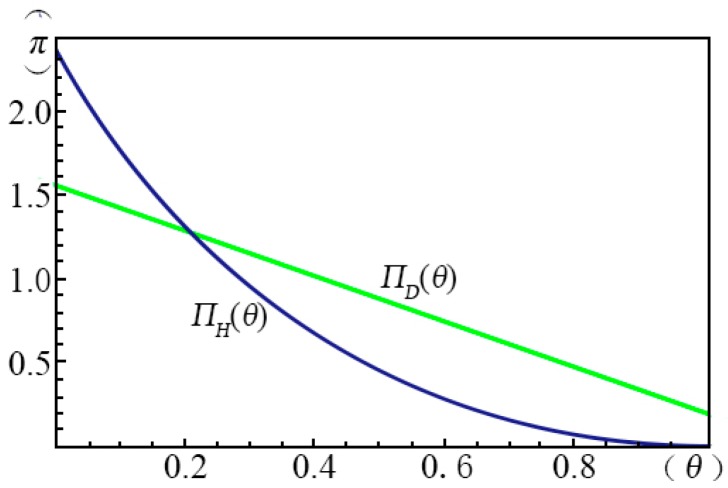
Comparison of the profits of the margin strategy and the intertemporal strategy as a function of θ (intercepted from Figure 9).

In Figure 9, we can also find that at $P\left(\alpha_{1},\xi_{1}\right)$, the *volume strategy* (*L*) has an obvious advantage. When θ is small (i.e., when the fraction of myopic consumers is large) the creator can earn the largest profits by applying the *intertemporal strategy* (*D*). This is because the myopic consumers will make the purchase decision instantly once they find that the surplus of the crowdfunding product is smaller than that of its substitute in the current stage. When α *and* ξ are fixed to other values, the shape of the curves of the profit functions of the four pricing strategies are similar to Figure 9, although there will be some translations and extensions. The fraction of the strategic consumers is influence by the concepts, education levels and advertising. Cumming, D.J. [5] used the data from Indiegogo, the second-largest reward-based crowdfunding platform worldwide. He observed that the success of green crowdfunding is more common in countries with lower individualism and educational background. This empirical study coincides with our results, as shown in Figure 10, the more myopic consumers have, the larger profit the creator will gain. Therefore, we recommend that the creator researches the market thoroughly before making the pricing decision. Not only should the creator investigate the fractions of high and low evaluating consumers and the gap between their valuations, but they should also take the current fraction of strategic consumers into account, especially this fraction among the applicable groups of the crowdfunding product.

## 6. Further Research

Our work provides a general framework for future study of crowdfunding pricing strategies under the existence of substitutes. There are some future research priorities. First, only two consumers were involved in our study. We will extend our model to cases where there are more consumers. Second, the current crowdfunding websites (e.g., Jingdong crowdfunding, etc.) provide batch discounts for heavy-buying consumers. This will be one of our future research directions. Finally, this paper inherited the two-period game setting approach from Ming Hu [18], which states that later-coming consumers can make their decisions based on the earlier-coming consumers. Nevertheless, it is well known that crowdfunding is a continuous mechanism, thus it is very possible that information asymmetry exists. Further research will also explore the pricing strategies given asymmetric information.

## 7. Conclusions

In recent years, crowdfunding has been regarded as a viable and popular alternative channel for entrepreneurs to fund their early stage businesses. In this paper, we studied green crowdfunding product pricing strategies when substitutes exist. When there are substitutes for the green crowdfunding product on the market, strategic consumers tend to choose the one that will give them the maximum consumer surplus according to their own valuations. Different consumers would have different valuations of one product, thus exhibiting different behavior in the purchase process. Through an equilibrium analysis, we found that when the fraction of high-type consumers is high, the margin strategy can make more profit for the creator. However, when the function of the substitute is better than the green crowdfunding product, menu pricing is almost not applicable. When the green crowdfunding product is twice as good as its substitute, menu pricing conversely has a great advantage. The higher the fraction of high-type consumers, the more different the consumer valuations, which enables the creator to make more profit. Generally speaking, when the fraction of high-type consumers and the gap between high and low valuations are both big, or both small, the traditional single pricing method shows its benefit.

Therefore, we suggest that the creator should investigate the differences between the consumers in the market first, before setting the pricing strategy. The green crowdfunding project should build their special green product or service based on the demand of the consumers, such as green food, green furniture, and so on. Simultaneously, the creator can carry out some green product promotion through new media such as video and pictures. Cumming D.J. [5] believed that successful green crowdfunding products are more likely to make use of soft information, including more galleries, video pitches, and long and better-worded project descriptions. For example, it is very attractive to invite some influential or famous stars to join in these green crowdfunding projects. Besides, the creator can choose the appropriate crowdfunding platform to increase the fraction of high-type consumers. Furthermore, the fraction of strategic consumers has a large influence on the total profits, thus this should have a large influence on the choice of pricing strategy. Thus, we recommend that the creator should also take the fraction of strategic consumers into account when making the market survey. Finally, the government should strengthen supervision and risk monitoring of crowdfunding platforms, to ensure that consumers will participate in green crowdfunding.

## Figures and Tables

**Table 1:** Symbols.

Symbol	Mean
$p_{1A}$	The price of *A* in the first stage (decision variable)
$p_{2A}$	The price of *A* in the second stage (decision variable)
*p*	The price of B in the first and second stage
$s_{ij}$	The consumer surplus of *j* (*j* = *A*, *B*) in the *i* (*i* = 1, 2) stage
